# 

**Published:** 1842-07

**Authors:** 


### CONTENTS OF No. XXVII.

OF THE

### British and Foreign Medical Review.

JULY, 1842.

---

### PART FIRST.—Analytical and Critical Reviews.

PAGE

ART. I.—Clinique Chirurgicale de l'Hôpital de la Pitié. Par J. LISFRANC . 1

Clinical Surgery of the Hospital of La Pitié. By J. LISFRANC.

ART. II.—A Treatise on Dislocations and Fractures of the Joints. By Sir A. COOPER, Bart., F.R.S. Edited by BRANSBY B. COOPER, F.R.S. . . 28

ART. III.—Traité des Fièvres Intermittents, Remittents, et Continués, des Pays Chauds, et des Contrées Marécageuses, suivi de Recherches sur l'Emploi Thérapeutique des Préparations Arsénicales. Par J. C. M. BOUDIN . 43
Treatise on Intermittent, Remittent, and Continued Fevers of Hot Climates and Marshy Countries, followed by Researches on the Therapeutic Employment of Arsenical Preparations. By J. C. M. BOUDIN.

ART. IV.—Medico-Chirurgical Transactions; published by the Royal Medical and Chirurgical Society of London. Second Series. Vol. VI. . . 49

ART. V.—Grundzüge einer neuen und wissenschaftlich begründeten Cranioscopie. Von CARL GUSTAV CARUS . . . . . . 65

Principles of a New and Scientifically-based Cranioscopy. By C. G. CARUS.

ART. VI.—Versuch einer Geschichte der Geburtshülfe. Von Dr. E. C. J. VON SIEBOLD, &c. Erster Band . . . . . . 80

An Essay towards a History of Midwifery. By Dr. E.C. J. VON SIEBOLD, &c. Vol.I.

ART. VII.—Nosologish-therapeutische Untersuchungen über die brandige Zerstörung durch Behinderung der Circulation des Blutes. Von CARL F. F. HECKER . 84
Nosological and Therapeutical Researches on Gangrene from Obstruction to the Circulation of the Blood. By F. F. HECKER.

ART. VIII.—1. Traité Pratique des Hernies, Déplacements et Maladies de la Matrice, &c. Par P. L. VERDIER . . . . 91

2. Leçons Cliniques sur les Hernies. Par J. F. MALGAIGNE . . ib.

3. A Treatise on Ruptures. By W. LAWRENCE, F.R.S. &c. . . . ib.

4. Traité de Pathologie Externe, et de Médecine Opératoire. Par AUG. VIDAL (DE CASSIS), &c. . . . . . . . ib.

ART. IX.—Quid faciant Ætas annique tempus ad frequentiam et diuturnitatem Morborum Hominis adulti, Inquisitio Medico-Statistica. Auctore E. FENGER, L.M. . . . . . . . 111
On the Influence of Age and Season on the frequency and duration of the Diseases of Adult Males. By E. FENGER.

ART. X.—Guy's Hospital Reports. No. XIII. October, 1841 . . 122

ART. XI.—Essais sur la Méthode Sous-cutanée, etc., précédés d'une Introduction Historique sur l'Origine et la Constitution de cette Méthode. Par le Dr. JULES GUERIN . . . . . . 136
Essays on the Subcutaneous Method of Operating, preceded by an Historical Introduction on its Origin and Nature. By Dr. J. GUERIN.

ART. XII.—A Manual of General Therapeutics, with Rules for Prescribing, and a Copious Collection of Formulæ. By D. SPILLAN, M.D. . . . 140

ART. XIII.—Zur Würdigung des Theophrastus von Hohenheim. Von Dr. KARL FRIED. HEIN. MARX . . . . . . 147

On the Real Merits of Theophrastus von Hohenheim [Paracelsus.] By Dr. MARX.

ART. XIV.—Lectures on the Diseases of the Urinary Organs. By Sir BENJAMIN C. BRODIE, Bart., F.R.S. . . . . . . 159

ART. XV.—1. Die Perkussion und Auskultation. Von Dr. JOSEPH SKODA . 173
Percussion and Auscultation. By Dr. JOSEPH SKODA.
2. A Practical Treatise on Auscultation. By MM. BARTH and ROGER. Translated, with Notes, by PATRICK NEWBIGGING, M.D. . . . ib.

4291

PAGE

Art. XVI.—Observations on Tuberculous Consumption; containing new views on the Nature, Pathology, and Cure of that Disease, being an attempt to found its Treatment on Rational Principles, deduced from Physiology and confirmed by extensive application. By J. S. Campbell, m.d. . . 181

Art. XVII.—The Gulstonian Lectures for 1842, on the Mutual Relation between Anatomy, Physiology, Pathology, and Therapeutics, and the Practice of Medicine. By Marshall Hall, m.d. f.r.s. &c. . . . 197

Art. XVIII.—De Eventu Sectionis Cæsareæ, etc. Auctore C. Kayser . . 199

Art. XIX.—1. Analekten über Kinderkrankheiten. Band III. und IV. . . 202

2. Von Mezler, Sammlung abseilesener Abhandlungen über Kinderkrankheiten. IX. Hefte . . . . . . . . ib.

Art. XX.—Die Lehre von den Zeichen, Ersheinungen und der Dauer der menschlichen Schwangerschaft, so wie von den Phänomenen einer überstandenen Geburt. Von W. F. Montgomery, a.m. m.d. m.r.i.a. . . 207

An Exposition of the Signs and Symptoms of Pregnancy, the Period of Human Gestation, and the Signs of Delivery. By W. F. Montgomery, a.m. m.d. m.r.i.a.

Art. XXI.—Principles of Surgery. By James Syme, f.r.s.e. . . 211

---

### Part Second.—Bibliographical Notices.

Art. I.—A Therapeutical Arrangement of the Materia Medica, or the Materia Medica arranged upon Physiological Principles, and in the order of the General Practical Value which remedial agents hold under their several denominations, and in conformity with the physiological doctrines set forth in the Medical and Physiological Commentaries. By Martyn Paine, m.d. a.m. 213

Art. II.—A Brief Commentary on Functional Derangement of the Stomach in Indigestion. By William Wynter, m.d. . . . . 215

Art. III.—Discourse on the Enlarged and Pendulous Abdomen, &c. augmented by a Dissertation on Gout, suggesting New Physiological Views of its Cause, &c. By Richard Frankum, Esq. . . . . . . ib.

Art. IV.—The First and Second Reports of the Medical Missionary Society in China; with Minutes of Proceedings, Hospital Reports, &c. . . 216

Art. V.—Dr. Hooper's Physician's Vade-Mecum; or Manual of the Principles and Practice of Physic. New Edition, considerably Enlarged and Improved, with an Outline of General Pathology and Therapeutics. By W. A. Guy, m.b. 217

Art. VI.—Observations on the Admission of Medical Pupils to the Wards of Bethlem Hospital, for the purpose of studying Mental Diseases. By J. Webster, m.d. ib.

Art. VII.—Interesting Facts connected with the Animal Kingdom; with some Remarks on the Unity of our Species. By John C. Hall, m.d. . . 218

Art. VIII.—Spinal and Nervous Diseases, Rheumatism, and Paralysis; or Cases and Observations illustrating an Improved Treatment. By J. H. Robertson, m.d. 219

Art. IX.—The Practice of Medicine; or a Treatise on Special Pathology and Therapeutics. By Robley Dunglison, m.d. . . . . . 220

Art. X.—The Anatomist's Vade Mecum; a System of Human Anatomy. By Erasmus Wilson . . . . . . . ib.

---

### Part Third.—Selections from the British and Foreign Journals.

### I. THE FOREIGN JOURNALS.

#### ANATOMY AND PHYSIOLOGY.

Dr. Budge's Physiological results of Extirpation of the Salivary Glands . 221
Professor Bochdalek on the Nerves of the Hard Palate . . . . 222
Dr. Stilling on the Anterior Columns of the Spinal Cord . . . ib.

PAGE
M. de Romane on the Fatty Substance of Milk . . . . . 222
Closure of the Nasal Fossæ without "Nasillement" . . . 223
M. Donne on the Origin, Mode of Formation, and End of the Blood-globules . ib.
Dr. Julius Budge on the Cause of the first Respiration after Birth . . ib.
Dr. Joseph Engel on Apoplexia Intermeningea . . . . ib.

### PATHOLOGY, PRACTICAL MEDICINE, AND THERAPEUTICS.

M. Landouzy on the Typhus at Rheims in 1833 and 1840 . . . 224
Dr. Briquet's Statistical Researches into the Etiology of Pulmonary Phthisis . 225
M. Max. Durand Fardel on a Peculiar Alteration of the Cerebral Substance . ib.
Dr. Petit on a Peculiar Matter secreted on the Hands of a Gouty Person . 227
Dr. Wagner's singular case of Suppuration of the Arachnoid . . ib.
M. Celestin Perrin on the employment of Sulphate of Alum in Angina Pharyngea 228
M. Recamier on the Delirium following Acute Meningitis, and its Treatment . ib.
M. Dichost's Extemporaneous Production of Milk . . . . 229
M. Bouchardat on the External Application of Croton Oil . . . ib.
Dr. Lüdicke on Tannin, as an Antidote for Strychnia . . . . ib.

### SURGERY.

Prof. Pecchiolli's Cure of Traumatic Tetanus by Division of the Nerve . 230
M. Velpeau on Fractures of the Lower Extremity of the Radius . . ib.
M. Radat on Dislocation of the Thumb . . . . . 231
M. Vidal on Artificial Anus. Closure of the Intestinal Canal by a Tumour . ib.
Signor della Fanteria on Reunion of two Fingers separated from the Hand . ib.
M. Velpeau on the Nature and Treatment of Erysipelas . . . 232
M. Trousseau on External Fistulæ of the Larynx . . . . ib.
Dr. C. Haller on Radical Cure of Reducible Inguinal Hernia . . ib.
M. Beynard on the Cure of Spina Bifida . . . . . 233
M. Charriere on Gilding of Surgical Instruments . . . . ib.
Prof. Rosas on a Case of Exophthalmus from Atheroma of the Orbit . . ib.
Louis Berg-de-Varsovic on the Operation for Hare-lip . . . 234
M. Begin on Hemorrhage after Lithotomy . . . . . ib.
Dr. C. L. Sigmund on the Radical Cure of Reducible Hernia . . . ib.
Dr. de Castella's Case of Varicose Tumour produced by Cancer of the Rectum . 235

### MIDWIFERY, AND DISEASES OF WOMEN AND CHILDREN.

Dr. C. Gnoli on the occurrence of confluent Smallpox in a Child before birth . ib.
M. Lisfranc's Experiments on the best Agent in Cauterizing Ulcerated Cervex Uteri ib.
Dr. Duntzer on Indentation of the Os Frontis of a new-born Infant . . 236
Dr. Schöller's Case in which the Placenta was retained after premature birth . ib.
Dr. Riecke on the Cure of Acute Hydrocephalus by Discharge of Water from the Ear 237

### MEDICAL STATISTICS.

Results of Revaccination in the Prussian Army in 1841 . . . ib.

---

### II. THE BRITISH JOURNALS, &c.

### ANATOMY AND PHYSIOLOGY.

Mr. R. W. Smith on Congenital Luxation of the Inferior Maxilla . . 238
Dr. Paton on the Spinal Cord in Cold-blooded Animals . . . ib.
Dr. Wright on the Physiology of the Saliva . . . . 239
Dr. Ranking on the Normal Dimensions of the Heart in the Adult . . ib.
Dr. Hall on the Male Influencing the Duration of the Fœtus in Utero ? . 240

### PATHOLOGY, PRACTICAL MEDICINE, AND THERAPEUTICS.

PAGE
Dr. WATTS on the Globules in Health and Disease . . . . 240
Dr. GRAVES's Diagnosis and Treatment of certain Cardiac Affections . . 241
Dr. STOKES on Cancer of the Lungs and Mediastinum . . . . 242
Drs. DRYSDALE and RUSSELL on the Pathology of Typhus . . . 243
Dr. J. Y. SIMPSON's Antiquarian Notices of Leprosy . . . . 244
Dr. ANDERSON on Valvular Diseases of the Heart . . . . . 245
Dr. PEEBLES on a Case of Hysterical Affection of the Eyes, with Closure of the Lids 246
Mr. WM. LYON on a Case of Hydrencephalocele . . . ib.
Mr. ALFRED SMEE on Violent Hysteria in a Man . . . . 247
Mr. JOSEPH BELL on Diabetes Mellitus: Cure (?) . . . . . ib.
Mr. GOODSIR on Vegetable Organisms ejected from the Stomach . . ib.
Dr. J. B. THOMPSON on Spontaneous Perforation of the Stomach . . 248
Dr. DUNNE's Two Cases of Glanders in the Human Subject . . . ib.

### SURGERY.

Dr. CHARLES CLAY's Case of Enlargement of the Nose treated successfully . . 249
Mr. TEALE's Five Cases of Stone treated by Lithotrity . . . . . ib.
Dr. DAWSON's Operation for Hare-lip performed on a Child four days old . 250
Mr. J. TOOGOOD on Dry Gangrene of the Arm. . . . . ib.
Air-douche of the Eustachian Tube . . . . . ib.
Mr. PARROTT on the Operation for Artificial Anus . . . . . ib.
Mr. TEALE on the Operation for Artificial Anus . . . . . 251
Dr. DONOVAN's Reduction of a Dislocation of Lower Jaw, 98 days after the accident 252
Dr. BARTON on the Seton in Chronic Disease of the Brain . . . ib.
Mr. HUNT on Subconjunctival Dislocation of the Lens . . . ib.
Mr. BULLEY on Rare Fracture of the Neck of the Thigh-bone . . . ib.
Dr. PRICHARD on Paracentesis of the Abdomen with the common grooved Needle 254
Mr. HIGGINS on Traumatic Tetanus: Cure . . . . . 254
Dr. OKE on the Spontaneous Obliteration of the Axillary Artery . . ib.
Mr. GRANTHAM's Case of Calculus in a female Child three years and a half old . 255
Mr. RUDDOCK on a Penetrating Wound of both Lungs: Recovery . . ib.
Mr. ALFORD on a Successful Case of Tracheotomy . . . . . ib.

### MIDWIFERY, AND DISEASES OF WOMEN AND CHILDREN.

Dr. SKIPTON's Case of Twins, with Union of the Bodies of the Children . . ib.
Mr. W. TAIT on the Birth of a Living Child on the 179th day . . 256
Mr. J. REID's Breech Case . . . . . . . ib.
Mr. WEST's Enquiry into the Results of Puncture of the Head in Chronic Hydrocephalus ib.
Mr. BRAINE on Artificial Premature Labour . . . . . 257
Mr. STANILAND's New Mode of accelerating Labour . . . . . ib.

### MEDICAL JURISPRUDENCE AND TOXICOLOGY.

Dr. HANDYSIDE's Remarkable Case of Suicide . . . . . ib.

G. FOWNES on Detection of Arsenic in Complicated Liquids . . . 258

---

### PART FOURTH.—Medical Intelligence.

Mr. PAGET's Report on Microscopical Anatomy and Physiology . . . 259
Medical Reform . . . . . . . 296
Dr. THOMSON's Report on Respiration and Animal Heat . . . 297

